# Mirizzi syndrome with an unusual type of biliobiliary fistula—a case report

**DOI:** 10.1186/s40792-015-0052-2

**Published:** 2015-06-17

**Authors:** Tsutomu Kawaguchi, Tadao Itoh, Kazuhiro Yoshii, Eigo Otsuji

**Affiliations:** Surgery and Gastroenterological Center, Rakuwakai Marutamachi Hospital, Kyoto, Japan; Division of Digestive Surgery, Department of Surgery, Kyoto Prefectural University of Medicine, Kyoto, Japan

**Keywords:** Mirizzi syndrome, Biliobiliary fistula, Surgery, Chronic cholecystitis

## Abstract

Gallstone obstruction of the cystic duct, resulting in chronic cholecystitis and pressure necrosis leads to the formation of biliobiliary fistula (BBF). We herein reported a case of Mirizzi syndrome (MS) with an unusual type of BBF (Corlette type I) that was successfully managed by a staged treatment strategy. The patient was diagnosed with a solitary gallstone, marked atrophy of the gallbladder, and BBF and underwent mucosal incineration of the atrophic gallbladder and simple closure, followed by extirpation of gallbladder. Although an optimal treatment strategy has not yet been established for MS with BBF because of its rarity and anatomical variations in fistulas, the current treatment strategy may be applicable. In conclusion, clinicians need to carefully diagnose and evaluate chronic cholecystitis in MS with BBF and adopt an optimal treatment strategy to avoid the complication associated with this disease.

## Background

Gallstone obstruction of the cystic duct, resulting in chronic cholecystitis leads to rare complication that is difficult to treat such as Mirizzi syndrome (MS) [1]. The subtype of this syndrome is associated with fistula in an adjacent organ such as the bile duct, duodenum, or transverse colon [1–6]. Although superior diagnostic modalities, such as magnetic resonance cholangiopancreatography (MRCP), multidetector computed tomography (MDCT), and endoscopic retrograde cholangiopancreatography (ERCP), have recently emerged, MS with fistula in an adjacent organ, especially the formation of biliobiliary fistula (BBF), is often diagnosed through intraoperative findings. The common bile duct or hepatic duct may be involuntarily injured during cholecystectomy for MS. Therefore, clinicians need to consider the potential complications of MS with BBF. Regarding surgical procedures for MS with BBF, previous studies reported that the optimal surgical procedure for MS with BBF was partial cholecystectomy with or without cholecystcholedochoduodenostomy or external choledochostomy by a drainage tube such as a T-tube drain [2, 7–10]. However, these procedures are considered invasive and slightly cumbersome. Therefore, more appropriate surgical procedures need to be adopted for MS with BBF. We herein reported a case of MS with an unusual type of BBF (Corlette type I) that was successfully treated with the combined approach of endoscopic nasobiliary drainage (ENBD) and the relatively simple procedure of bile duct repair.

## Case presentation

**Fig. 1:**
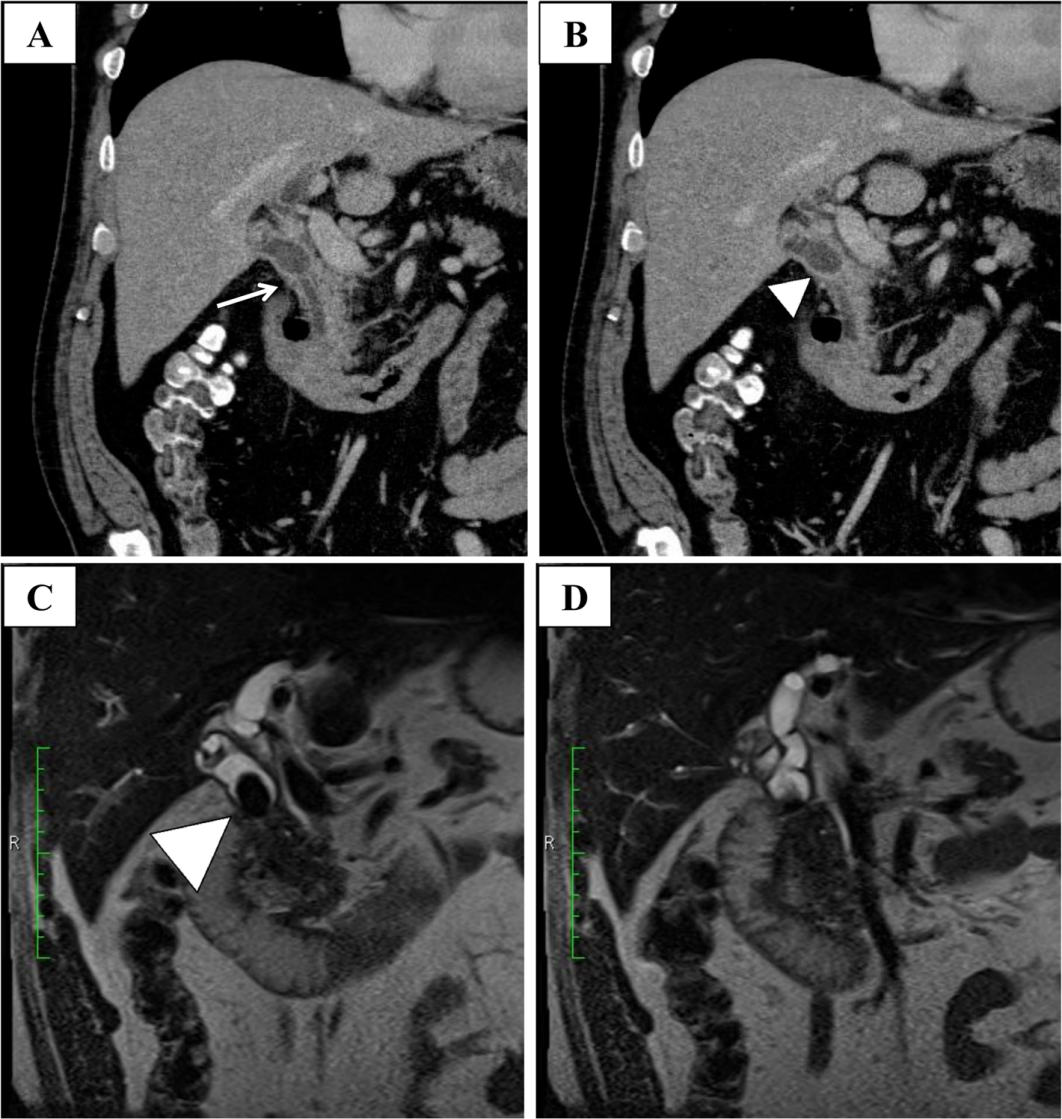
Preoperative diagnosis on MDCT and MRCP. Preoperative MDCT revealed mild dilation of the common bile duct (**a**) and a coalesced gallbladder with the bile duct (*arrow*), suggesting biliobiliary fistula (*small arrow head*) (**b**). Preoperative MRCP showed a solitary gallstone in the gallbladder (**c**, *large arrow* head), dilation of the intrahepatic bile duct, and biliobiliary fistula (**d**)

**Fig. 2:**
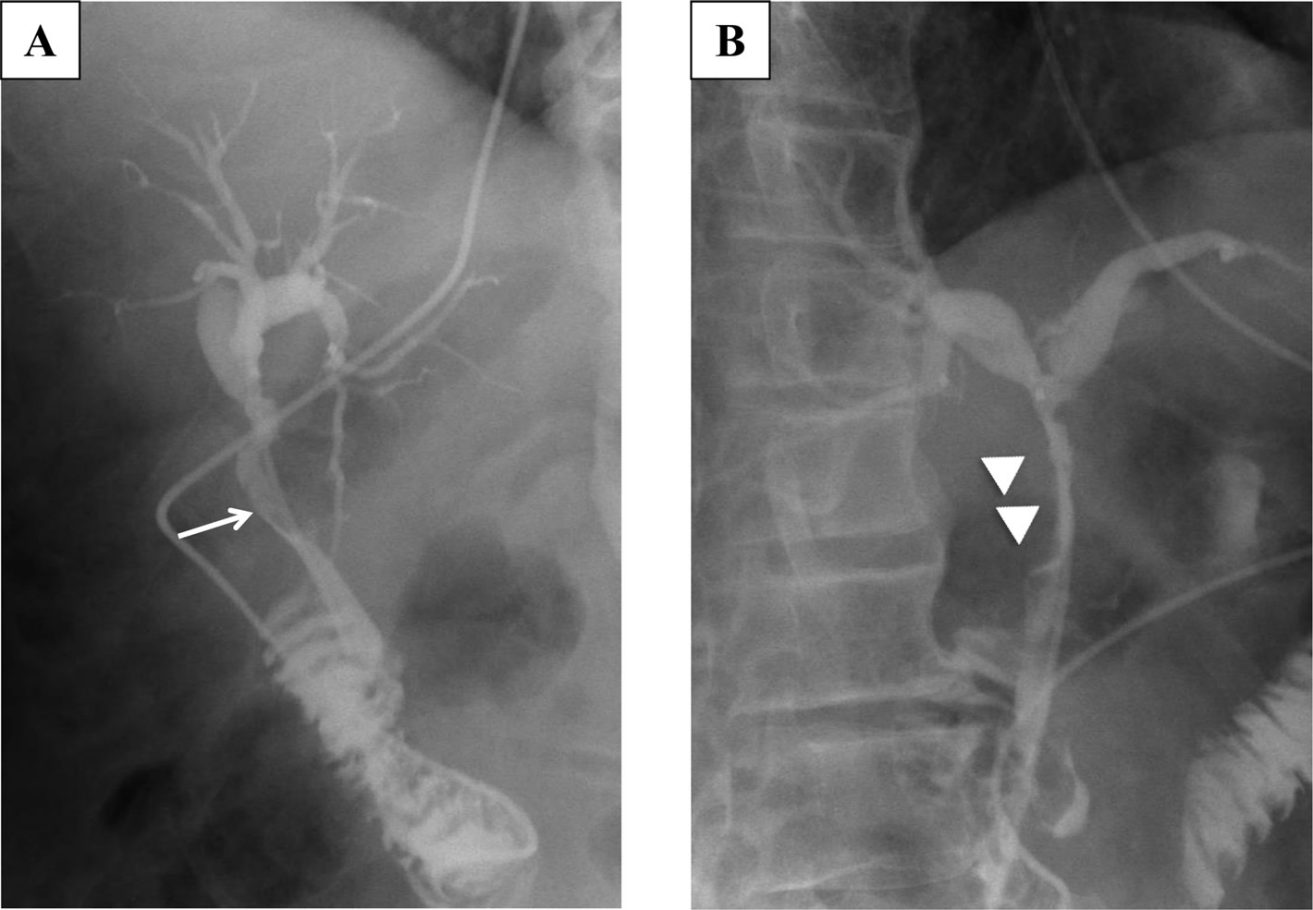
Preoperative cholangiography through an ENBD tube. Preoperative cholangiography through the ENBD tube may show bile duct compression by the gallstone. Inflow of the contrast media into the atrophied gallbladder (*arrow*, **a**) and compressed bile duct (*arrow head*, **b**)

A 57-year-old man was admitted to our hospital with severe epigastric pain, jaundice, and high fever. On admission, his body temperature was 38.5 °C, heart rate was more than 100 beats/min, and blood pressure was 165 mmHg, suggesting systematic inflammatory response syndrome. Laboratory data showed a white blood cell count of 12,600/mm [3], C-reactive protein level of 0.79 mg/dl, total bilirubin level of 4.7 g/dl, aspartate aminotransferase (AST)/alanine aminotransferase (ALT) level of 471/292 U/l, and gamma-glutamyl transpeptidase (γ-GTP) level of 1452 U/l. These data suggested acute obstructive suppurative cholangitis (AOSC) due to obstruction of the bile duct. Abdominal MDCT revealed mild dilation of the common bile duct and a coalesced gallbladder with the bile duct (Fig. 1) suggesting MS with BBF. MRCP also detected a solitary gallstone at the most distal site of the dilated bile duct (Fig. 1). The patient underwent ERCP including endoscopic sphincteropapillotomy (EST), followed by ENBD (Fig. 2), and the severe inflammation status and obstructive jaundice were immediately improved. Preoperative bile cytology was performed, and no malignant epithelial cells were detected. Therefore, radical surgery was planned to remove the solitary gallstone and perform choledochoplasty.

**Fig. 3:**
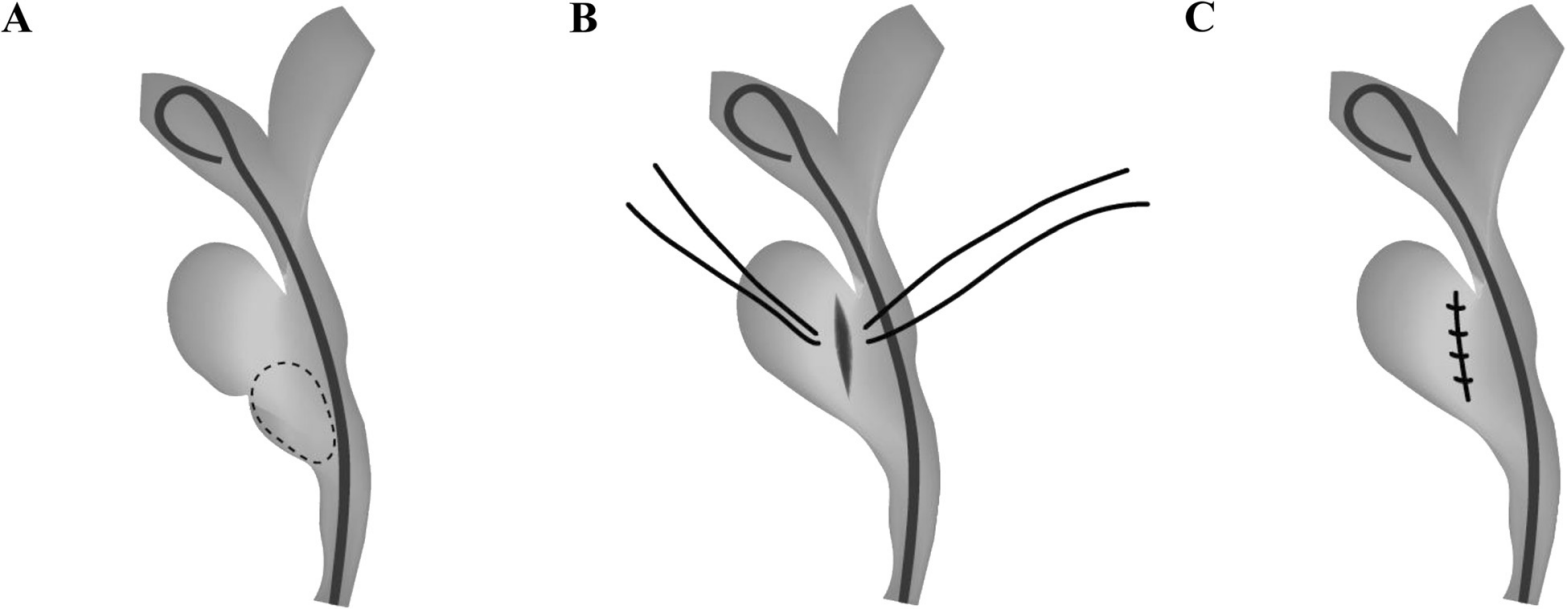
Schema of the intraoperative findings. This schema showed intraoperative findings of the case. The *dashed line* is solid gallstone in the neck of the gallbladder (**a**). Schema of the operative procedure (**b**, **c**)

**Fig. 4:**
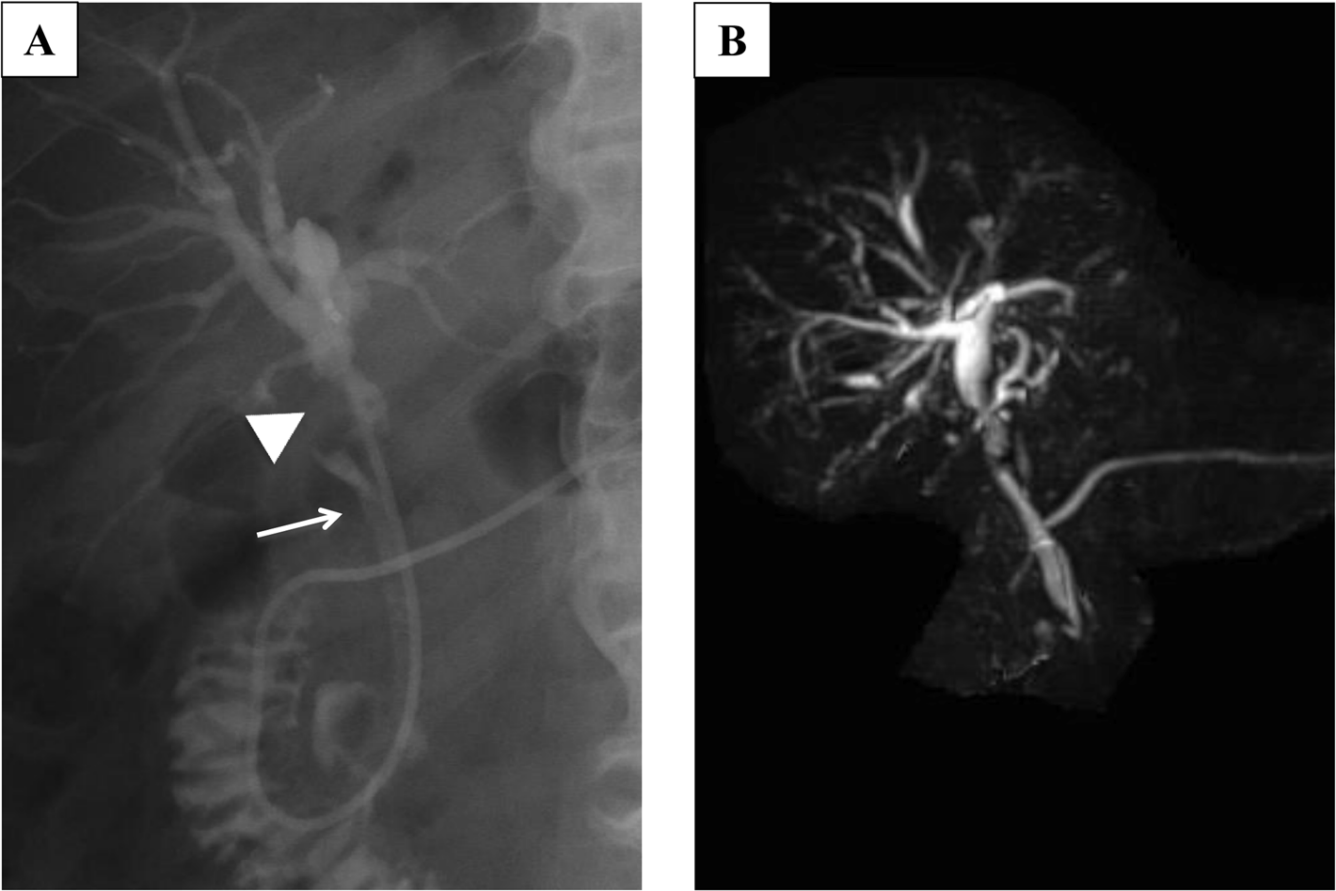
Postoperative cholangiography through an ENBD tube and postoperative MRCP. Postoperative cholangiography through the ENBD (1 week after surgery, **a**) and MRCP (1 month after surgery, **b**) showed profluent flow in the bile duct, no stagnant fluid in the remnant gallbladder, and no dilation of the upper bile duct (*arrow heads* surgical repair site, *arrow* cystic duct)

Operative findings revealed BBF with marked atrophy of the gallbladder and a solitary gallstone, and mucosal incineration of the atrophic gallbladder and simple closure guided by the ENBD tube, followed by extirpation of gallstone were performed. Intraoperative cholangio-endoscopy via the true fistula showed that the mucosae of the common bile duct and hepatic bile duct were flat and smooth, suggesting no malignancy. The patient showed a good postoperative course, and profluent flow in the bile duct was confirmed by postoperative direct cholangiography and MRCP (Fig. 3). We herein described a rare case of MS with BBF type I with a markedly atrophied gallbladder that was successfully managed by endoscopic diagnostic therapy followed by a minimally invasive surgical procedure (Fig. 4).

## Discussion

Gallstone obstruction of the cystic duct, resulting in chronic cholecystitis leads to rare complications that are difficult to treat such as MS [1]. The subtype of this syndrome is associated with fistula in an adjacent organ such as the bile duct, duodenum, or transverse colon [1–6]. Gallstone obstruction of the cystic duct, resulting in chronic cholecystitis and pressure necrosis leads to the formation of BBF [1, 3–5]. Another risk of the disease is adhesion of gallbladder and bile duct due to cholecystitis. On the other hand, differential diagnosis of bile duct malignancy is one of the most important problems in clinical practice of handling MS. In the current case, preoperative bile cytology and intraoperative cholangio-endoscopy demonstrated no malignancy of bile duct.

Although superior diagnostic modalities, such as MRCP, MDCT, and ERCP have recently emerged, MS with BBF is often diagnosed based on intraoperative findings. Therefore, clinicians need to consider the potential complications of BBF. A clearer understanding of precise anatomical variations and the optimal treatment strategy for this rare and difficult to treat complication of MS is important in order to avoid short- and long-term morbidities such as bile duct injury.

Recent studies reported that the optimal surgical procedure for MS with BBF may be partial cholecystectomy with or without cholecystcholedochoduodenostomy or external choledochostomy by a drainage tube such as a T-tube drain [2, 7–10]. In the present case, cholecystectomy could not be performed because of marked atrophy of the gallbladder, and cholecystcholedochoduodenostomy or external choledochostomy may not be suitable. One of the potential limitations of the surgical therapies for MS with BBF is ductal stenosis due to injury to the common bile duct. In the present case, preoperative imaging including CT, MPCP, and ERCP revealed a markedly atrophied gallbladder as well as a solitary gallstone. Therefore, extirpation of the gallstone and repair of the biliary tract guided by the ENBD tube was planned and adopted as a treatment for BBF with marked atrophy of the gallbladder. Mucosal incineration of the atrophied gallbladder and simple closure guided by the ENBD tube, followed by extirpation of gallstone were performed, and this simple procedure of minimally invasive and radical surgical repair of the bile duct was successful.

## Conclusions

Clinicians need to carefully diagnose and evaluate chronic cholecystitis in Mirizzi syndrome with BBF and adopt an optimal treatment strategy that includes a surgical procedure to avoid the complication associated with this disease.

## Consent

Written informed consent was obtained from the patient for the publication of this case report and any accompanying images. A copy of the written consent is available for review by the Editor-in-Chief of this journal.

## Abbreviations

BBF: biliobiliary fistula
ERCP: endoscopic retrograde cholangiopancreatography
ENBD: endoscopic nasobiliary drainage
MRCP: magnetic resonance cholangiopancreatography
MDCT: multidetector computed tomography
